# Spanish adaptation of the Burden Assessment Scale in family caregivers of people diagnosed with borderline personality disorder

**DOI:** 10.1186/s40479-023-00211-6

**Published:** 2023-02-20

**Authors:** Joaquín García-Alandete, Isabel Fernández-Felipe, Sara Fonseca-Baeza, Irene Fernández, Sandra Pérez, José H. Marco, Verónica Guillén

**Affiliations:** 1 Department of Personality, Evaluation and Psychological Treatments, University of Valencia, Avda. Blasco Ibáñez, 21, 46010 Valencia, Spain; 2 Department of Basic and Clinical Psychology and Psychobiology, University Jaume I, Avda. Vicent Sos Baynat, S/N. 12071, Castellón de La Plana, Spain; 3 Department of Behavioral Sciences Methodology, University of Valencia, Avda. Blasco Ibáñez, 21. 46010, Valencia, Spain; 4 CIBER Physiopathology, Obesity and Nutrition (CB06/03), Carlos III Health Institute, Av. Monforte de Lemos, 3-5. Pabellón 11. Planta 0, 28029 Madrid, Spain

**Keywords:** Family caregivers, Burden, Burden Assessment Scale, Borderline personality disorder, Psychometric properties

## Abstract

**Background:**

Caregiving is a strong source of stress and leads the family caregiver to experience the burden of being responsible for the care of a severely mentally ill family member. The Burden Assessment Scale (BAS) assesses burden in family caregivers. This study aimed to analyze the psychometric properties of the BAS in a sample of family caregivers of people diagnosed with Borderline Personality Disorder (BPD).

**Methods:**

Participants were 233 Spanish family caregivers (157 women and 76 men aged between 16–76 years old, *M* = 54.44, *SD* = 10.09) of people diagnosed with BPD. The BAS, the Multicultural Quality of Life Index, and the Depression Anxiety Stress Scale-21 were used.

**Results:**

An exploratory analysis resulted in a three-factor 16-item model (Disrupted Activities; Personal and Social Dysfunction; Worry, Guilt, and Being Overwhelmed) with an excellent fit (χ^2^(101) = 56.873, p = 1.000, CFI = 1.000, TLI = 1.000, RMSEA = .000, SRMR = .060), good internal consistency (ω = .93), a negative correlation with quality of life, and a positive correlation with anxiety, depression, and stress.

**Conclusion:**

The model obtained for the BAS is a valid, reliable, and useful tool for assessing burden in family caregivers of relatives diagnosed with BPD.

## Introduction

Caring for people diagnosed with a severe mental disorder (SMD) usually falls on their relatives, for whom such responsibility is a strong source of stress and leads them to experience burden (e.g. [26]). Burden includes two dimensions, objective (financial problems, limitations on personal activity, household disruption, and social interactions) and subjective (shame, stigma, guilt, resentment, grief, and worry) (e.g. [35]), and can be conditioned by many variables (e.g. [40, 42]).

### Family caregiving of people diagnosed with borderline personality disorder

Bailey and Grenyer [4], in a review of several studies about burden and support needs of carers of people with borderline personality disorder (BPD) [16, 18–20, 39], found that (1) the caregivers experienced elevated objective and subjective burden, grief, impaired empowerment, and mental health problems, including depression and anxiety, and (2) scores on both objective and subjective burden were half a standard deviation above the mean, compared to caregivers of inpatients with other SMD (e.g. schizophrenia). These authors concluded that caring for a relative diagnosed with BPD seems to be harder and more burdensome than caring for a family member with another SMD, due to the psychopathological characteristics of BPD [2].

Kay et al. [27] found that family members who were caring for their relatives diagnosed with BPD: (1) described their relatives as experiencing emotional, behavioral, interpersonal, and self-dysregulation problems; (2) expressed negative feelings towards their relatives; (3) experienced social humiliation, financial strain, and marital discord; (4) wanted to move forward and improve their mental health; (5) experienced a challenging process of adaptation and coping; and (6) experienced a quest for harmony and integration. These authors concluded that being knowledgeable about the relative’s mental disorder is quite important because it can empower the caregiver. This conclusion, however, contrasts with the results obtained by Hoffman et al. [18], who found that the greater the knowledge about the BPD, the higher the level of family members’ burden, depression, distress, and hostility toward their relatives diagnosed with BPD. In this regard, “there is a consensus on the necessity that relatives of BPD subjects should have the opportunity to receive state-of-the-art, evidence-based information on BPD and its available treatments, in order to destigmatize the BPD diagnosis as well as the role of the family in BPD development” ([14], 3) [18–20].

Jørgensen et al. [26] found that higher BPD severity at the end of mentalization-based treatment (one year) in adolescents predicted family caregiver burden, and that biological mothers could be more burdened than other types of caregivers. These authors suggested that caregivers, especially biological mothers, of adolescents with more severe levels of BPD could be particularly vulnerable to feelings of burden and, therefore, need more support [8].

### The Burden Assessment Scale

Reinhard et al. [36] stated that the burden measures proposed up to that time had some limitations that underrepresented the burden of families who do not live with their mentally ill relative. According to these authors, there was a need for a measure of burden that was independent of the living situation of the ill family member and that focused on specific caregiver consequences. Likewise, these authors argued that it was necessary to have a brief, valid, and reliable assessment tool that focuses on specific objective and subjective caregiver consequences, in order to test the effectiveness of programs designed to alleviate the burden on family caregivers of people diagnosed with SMD. With these issues in mind, Horwitz and Reinhard [21] developed the Burden Assessment Scale (BAS), a 19-item scale for assessing both subjective and objective burden in family caregivers of people diagnosed with SMD.

Several studies have analyzed the psychometric properties of the BAS, obtaining different results for both the number of factors and the items included in each factor (Table 1). Horwitz and Reinhard [21], Reinhard et al. [36], and Aydemir et al. [3] obtained a five-factor model. Murdoch et al. [33] obtained a four-factor model. Hunger et al. [22] found that the model with the best fit had four correlated factors, and it was obtained from Reinhard et al.’s [36] study. Ivarsson et al. [23] and Kwak et al. [29] obtained a three-factor model, and Guada et al. [17] obtained a two-factor model. Hunger et al. [22] used confirmatory procedures, whereas the other studies used principal component analysis.

**Table 1:** Models proposed for the BAS

Study	Sample	Factorial procedures, factors obtained, and internal consistency of the BAS	Validity of the BAS and other significant results
Horwitz and Reinhard [21]	94 family members of severely mentally ill adults who participated in a community aftercare program in New Jersey (The Club)	1. Financial Distress (items 1 and 6) [OB] 2. Personal Activity (items 2–5) [OB] 3. Negative Effects on Social Interactions (items 7–10) [OB] 4. Feelings, Attitudes, and Emotions (items 11–16, and 19) [SB] 5. Worry (items 17 and 18) [SB]	
Reinhard et al. [36]	Sample 1 (Club): the sample used in Horwitz and Reinhard [21] study Women: 77 (81.9%) Men: 17 (18.1%) *M*_age_ = 58 Sample 2 (DMH&H): 94 family members of mentally ill adults who participated in a study of the New Jersey’s DMH&H Women: 64 (68.1%) Men: 29 (30.8%) *M*_age_ = 56.5	PCA with Varimax rotation Sample 1 (Club): 1. Disrupted Activities (items **2**, 4–8, and **15**) [OB] 2. Personal Distress (items 1, 9–11, 14, **15**, and 19) [SB] 3. Guilt (items **2**, 13, and 17) [SB] 4. Time Perspective (items 16 and 18) [SB] 5. Worry (items 3, 12 and **15**) [SB] Sample 2 (DMH&H): 1. Disrupted Activities (items 3–8) [OB] 2. Personal Distress (items 10, 11, 14, and 15) [SB] 3. Time Perspective (items 16, 18, and 19) [SB] 4. Guilt (items 12, 13, and 17) [SB] 5. Basic Social Functioning (items 2 and 9) [OB] Whole BAS α = .89 (Sample 1) and .91 (Sample 2)	Burden was higher for DMH&H family members than for Club family members Families in both samples: 1.- Reported the greatest average burden from their worry about the future and unrelenting grief 2.- Considered missing days at work, friction with neighbors, and guilt for causing the illness as least burdensome A lower score on the BAS was obtained over the six-month period after an intensive family support services program (the ill family member’s age and diagnosis were not predictive, nor was the involvement of the ill family member’s mother) Differential reduction in burden could be explained by service use: The more types of services received and the more single family contacts, the greater the reported reduction in family burden Families with more members involved in intensive family-support services are less likely to experience reductions in burden
Ivarsson et al. [23]	256 Swedish caregivers for individuals with SMDs Women: 82 (32%) Men: 174 (68%) Age range 18–81 years (*M* = 39.3, *SD* = ns)	PCA with Varimax rotation (minimum λ > .40): 1. Activity Limitation (items 1–8, and 15; α = .88) 2. Worry and Guilt (items 12, 13, 16–18; α = .73) 3. Social Strain (items 9–11, 14, and 19; α = .75) Whole BAS α = .90	The greatest perceived burden in caregivers: worry about the future The least perceived burden in caregivers: friction with others The caregivers experienced: 1. More activity limitations in relation to the youngest clients, those who had an elementary educational level, and those who lived with a partner 2. More feelings of worry and guilt in relation to the younger clients than to the older ones 3. More social strain in relation to the female clients and those who had an elementary educational level
Aydemir et al. (2012)^a^ [3]	100 Turkish volunteers who were caregivers of outpatients diagnosed with SMDs (schizophrenia, bipolar disorder, major depression, and anxiety) Women: 44 (44%) Men: 56 (56%) Age: *M* = 41.9, *SD* = 15.1	PCA with Varimax rotation (minimum λ > .40): 1. Limitations in Daily Life (9 items) 2. Worry for the Patient (5 items) 3. Negative Emotions (2 items) 4. Disruption in Activities (2 items) 5. Losses of the Caregiver (1 item) Whole BAS α = .89	Correlation with the Perceived Family Burden *r* = .49 Correlation with the Zarit Caregiver Burden Scale, *r* = . 61
Guada et al. [17]	106 family caregivers (86.2% women) of people diagnosed with schizophrenia 94 participants were African-American Participants were living in Los Angeles, USA Age range 18–80 years old Age *M* = 47 years old	PCA with Varimax rotation (limited to 2 factors): 1. Emotional Reactions of Caregiving (Items 9–19) [SB] 2. Daily Impacts of Caretaking (Items 1–8) [OB]	
Kwak et al. [29]	256 Korean family caregivers (84 men, 172 women) of people diagnosed with schizophrenia Age range 21–78 (*M* = 54.4, *SD* = 11.2)	Reliability and validity of the Korean version of the BAS (K-BAS) PCA with Varimax rotation (minimum λ = .40): 1. Activity Limitations (Items 1–8) 2. Social Strain (Items 9–11, 14–16, and 19) 3. Feelings of Worry and Guilt (Items 12, 13, 17, and 18) K-BAS α = .91 K-BAS test–retest reliability = .86, *p* < .001	Convergent validity: 1. Correlation between the K-BAS and the FBS: r = .80, *p* < .001 Divergent validity: 1. Correlation between the K-BAS and KDAI-10:* r* = -.21, *p* = .001 2. Correlation between the K-BAS and SUMD-K: *r* = -.02, *p* = .796
Murdoch et al. [33]	Canadian family caregivers of 300 children and adolescents diagnosed with SMD seeking services within a major city	PCA with Varimax rotation (minimum λ = .40): 1. Role Restriction (Items 1–7) 2. Family Impact (Items 8, 9, 14, and 15) 3. Public Embarrassment (Items 10, 11, and 19) 4. Guilt and Worry (Items 12, 13, 16, and 17) SB: Items 1–10 OB: Items 11–18 (although Item 18 did not load in any factor, because it loaded < .40 in all four factors)	
Hunger et al. [22]	215 relatives (72% women, 28% men) of German mentally ill people Age range 18–77 (*M* = 32, *SD* = 14)	CFA (Maximum-Likelihood method) Authors tested several models for the BAS. Model with the best fit was one with four correlated factors: 1. Disrupted Activities (Items 1–8) 2. Personal Distress (Items 9–11, 14, and 15) 3. Time Perspective (Items 16, 18, and 19) 4. Guilt (Items 12, 13, and 17) Internal consistency: Whole BAS, α = .92 (95% CI [.90, .93]) Subscales, α from .64 to .92 (95% CIs [.55 to. 92])	

*Note*. *PCA* Principal Component Analysis, *CFA* Confirmatory Factor Analysis, *DMH&H* Division of Mental Health & Hospitals, *KDAI-10* Korean version of Drug Attitude Inventory-10, *SUMD-K* Korean version of the Scale to Assess Unawareness of Mental Disorder, *FBS* Family Burden Scale, *[OB]* Objective Burden, *[SB]* Subjective Burden, *ns* not stated

^a^It was not possible to offer the items included in each factor because that information was not available in the paper (neither in the body of the text nor in the corresponding table is the composition of the factors adequately given)

In these studies, the internal consistency of the BAS ranged from questionable, as in the case of the factor Guilt (α = .64) in Hunger et al.’s [22] study, to good, α > .90. The BAS also showed good test–retest reliability in Kwak et al.’s [29] study, *r* = .86, *p* < .001. Likewise, in Aydemir et al. [3] and Kwak et al. [29] studies, the BAS showed divergent and/or convergent validity.

Some of these studies did not meet the requirements for accepting the result of the factor analyses performed. Horwitz and Reinhard [21] accepted two factors that included only two items each: Financial Distress included items 1 (Financial problems) and 6 (Upset household routine), and Worry included items 17 (Worry makes illness worse) and 18 (Worry about the future). Reinhard et al. [36] accepted items 2 (Missed work/school) and 15 (Felt trapped) in several factors in the model obtained with The Club sample. Similarly, these authors accepted one factor (Time Perspective) that included only items 16 (Upset about relative’s change) and 18 (Worry about the future), and they did not indicate in which factor item 1 (Financial problems) should be included (this was probably because this item loaded < .40 in the five factors they found) in the model obtained for the BAS with the DMH&H sample. Aydemir et al. [3] accepted two factors (Negative Emotions and Disruption in Activities) that only included two items, and they accepted one factor (Loss of the Caregivers) that only contained one item.

These results suggest the desirability of further investigating the structure of the BAS, an instrument that is widely used to assess the burden of family caregivers of people diagnosed with SMD.

### The present study

This study aimed to analyze the psychometric properties of the BAS in a sample of Spanish caregivers of people diagnosed with BPD, concretely to test the underlying factors of that scale and the fit of the obtained model using exploratory and confirmatory procedures respectively, as well as its internal consistency and construct validity. To our knowledge, this is the first study to analyze the psychometric properties of the BAS in the Spanish population.

## Method

### Participants

**Fig. 1:**
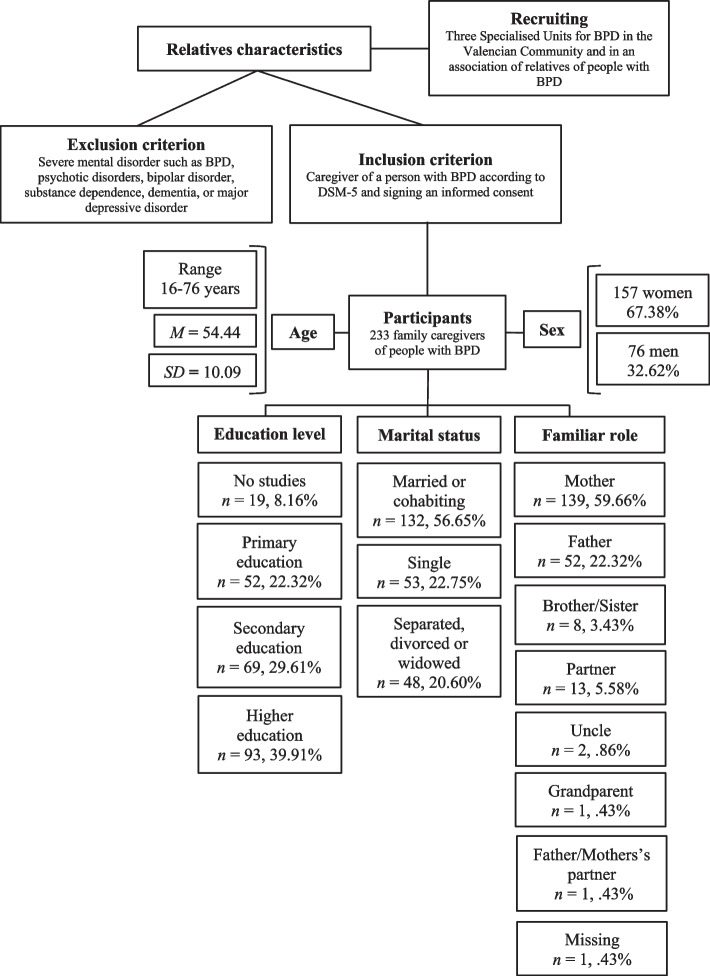
Flowchart of participants’ sociodemographic characteristics

Participants were 233 family caregivers of people diagnosed with BPD who were receiving psychological treatment in three Specialized Units for Personality Disorders and from an Association of Relatives of people with BPD in Spain (Fig. 1). The sample was collected over a period of three years (2018–2021). To be part of the research, the inclusion criteria were (a) being a caregiver of a relative with a diagnosis of BPD according to the Diagnostic and Statistical Manual of Mental Disorders [2] and (b) agreeing to and signing an informed consent regarding their voluntary participation in the study and the confidential treatment of their data. Exclusion criterion was to be diagnosed with an SMD, such as BPD, psychotic disorder, bipolar disorder, substance dependence, dementia, or major depressive disorder.

### Instruments

#### Burden Assessment Scale (BAS; [36])

The BAS is a 19-item scale that assesses both the subjective and objective burden of caregiving within the past six months. Subjective burden includes emotions, attitudes, and concerns associated with the caregiver role; objective burden covers observable aspects such as reduced personal time or financial problems. Answers are coded on a four-point Likert scale (1 = Not at all; 4 = A lot). The higher the score the higher level of caregiver burden. Internal consistency of the BAS in this study was ω = .93, 95% CI [.91, .94].

#### Multicultural Quality of Life Index (MQLI; [32])

We used the Spanish adaptation [31]. The MQLI is a 10-item scale that assesses physical and emotional well-being, self-care, occupational and interpersonal functioning, socio-emotional and community support, personal and spiritual fulfillment, and an overall perception of quality of life. Answers are coded on a 10-point Likert scale (1 = Bad,10 = Excellent). Internal consistency of the MQLI in this study was ϖ = .91, 95% CI [.90, .93].

#### Depression Anxiety Stress Scale-21 (DASS-21; [30])

We used the Spanish adaptation [13]. The DASS-21 assesses self-perceived physical and subjective symptoms of anxiety, depressive feelings, and behavioral manifestations of stress. Responses are coded on a Likert scale (0 = It did not happen to me,3 = It happened to me most of the time). In this study, internal consistency estimates were ω = .88, 95% CI [0.85, 0.90] for anxiety, ϖ = .90, 95% CI [.88, .92] for depression, and ω = .89, 95% CI [.87, .92] for stress.

### Statistical analyses

First, descriptive statistics, corrected item-total correlations, and McDonald’s ω change if an item was dropped from the BAS were calculated in the whole sample (*n* = 233). In addition to McDonald’s ω, the Average Interitem Correlation (AIC) of the BAS was analyzed (according to Clark and Watson [5], the AIC score should be between .15 and .50).

Second, an Exploratory Factor Analysis (EFA) using Weighted Least Squares (Bartlett’s test showed inequality of variances) with Oblimin rotation was performed in randomized Subsample 1 (*n* = 114, 48.93%) [7] in order to obtain a model for the BAS. The Measure of Sampling Adequacy (MSA) was calculated (a score of .50 is an acceptable limit for retaining a variable in the EFA, and a score of .80 is meritorious) (e.g. [41]). To assign an item to a factor, the factor loading had to be ≥ .40. To accept a factor in the model, it had to include at least three items with a loading ≥ .40. If an item had a factor loading ≥ .40 on two or more factors, the factor with a loading difference of ≥ .05 from the rest of the factors was chosen.

Third, a CFA of the model obtained for the BAS was carried out in randomized Subsample 2 (*n* = 119, 51.07%) [43]. Because Mardia’s coefficient was > 5 and data were ordinal, Diagonally Weighted Least Squares (DWLS) and robust methods were used [38]. Fit indices used were the Chi-Square (χ^2^), the Comparative Fit Index (CFI) and the Tucker-Lewis Index (TLI) (a value ≥ .90 suggests an acceptable fit, and a value ≥ .95 suggests a good model fit), the Root Mean Square Error of Approximation (RMSEA) and the Standardized Root Mean Square Residual (SRMS) (a value ≤ .08 suggests an acceptable model fit, and a value ≤ .05 suggests a good model fit) (e.g. [28]).

Fourth, to determine both the concurrent and divergent validity of the model for the BAS obtained in this study, the correlations with the MQLI and DASS-2 were analyzed using Spearman’s rho (ρ) and interpreted according to Cohen [6]. To carry out all the statistical analyses mentioned in this section, the JASP 0.16.1 software [24] was used.

### Procedure

An independent translator translated the scales above described from English to Spanish. Then, back-translation was carried out by another independent translator. Both translators were fluent speakers of both languages. Then, the authors of this study reviewed both the English and Spanish versions of each scale, which resulted in common format translations. The translated version of each scale was once again reviewed by an independent specialist in psychopathology. The last reviewed version of each scale was used in this study.

Participants were informed about the nature of this study, the treatment of the data, and the voluntary nature of their participation. Diagnostic interviews using the clinician version of the Structured Clinical Interview for DSM-5 (SCID-5) [12] were conducted in a clinical setting by psychologists with more than 10 years of experience in the assessment and treatment of mental disorders. Participants that met the inclusion criteria signed the informed consent form and completed the assessment protocol.

## Results

### Descriptive statistics of the BAS

Table 2 shows the descriptive statistics of the BAS. All the corrected item-total correlations were > .40 [.416, .718]. The mean for item 10 was 1.51, and this item was included in Factor 2. The mean score on item 1 was low (*M* = 1.92). The highest mean score was on item 18 (*M* = 3.70), close to the maximum of the scale. The mean for item 19 (*M* = 2.70) was comparable to that of other BAS items that were included in any factor (e.g. items 16 and 17, among others). Both the mean and kurtosis of item 18 stood out.

**Table 2:** Descriptive statistics of the BAS in the whole sample

BAS item	*M*	*SD*	*Sk*	*K*	ω if item dropped	Corrected *r*_(item-total)_
1. Financial problems	1.92	1.15	.575	-.896	.93	.433
2. Missed work/school	2.00	1.14	.347	-.991	.92	.560
3. Difficulty concentrating	2.95	1.01	-.585	-.668	.92	.698
4. Change personal plans	2.71	1.16	-.333	-1.218	.92	.680
5. Reduced leisure time	2.82	1.07	-.416	-1.005	.92	.704
6. Upset household routine	2.86	1.06	-.517	-.803	.92	.718
7. Less time for friends	2.67	1.10	-.291	-1.161	.92	.665
8. Neglected family’s needs	2.49	1.02	-.106	-1.023	.92	.713
9. Family friction	2.88	1.01	-.449	-.811	.92	.614
10. Friction with others	1.51	.99	1.081	.497	.93	.512
11. Embarrassed	2.11	1.20	.249	-1.071	.92	.649
12. Guilty for not helping enough	2.54	1.13	-.073	-1.130	.92	.569
13. Guilty for causing illness	2.29	1.19	.085	-1.164	.92	.590
14. Resented demands	2.12	1.11	.096	-.843	.92	.635
15. Felt trapped	2.67	1.17	-.354	-1.031	.92	.711
16. Upset about relative’s change	2.76	1.11	-.474	-.765	.92	.624
17. Worry about making illness worse	2.67	1.10	-.281	-1.010	.92	.578
18. Worry about the future	3.70	.64	-2.594	8.014	.93	.481
19. Stigma upsetting	2.75	1.11	-.406	-.952	.93	.416

*Note*. *N* = 233. Standard error of skewness = .159; Standard error of kurtosis = .318

As indicated above, the BAS showed good internal consistency, ω = .93, 95% CI [0.91, 0.94]. The AIC = .399, 95% CI [.351, .444] suggested that the items of the BAS were reasonably homogenous and contained enough unique variance to avoid being isomorphic with each other [34].

### Exploratory factor analysis of the BAS

All the MSAs for the BAS items were > .800, and the MSA for the whole BAS was 0.900. The solution showed a three-factor model for the BAS: Factor 1 contained 7 items (2–8), Factor 2 contained 6 items (9–11 and 14–16), and Factor 3 contained 3 items (12, 13, and 17) (Table 3). Items 1, 18, and 19 on the BAS loaded < .40 in all of the factors obtained. These factors showed a good internal consistency: whole scale, ϖ = 0.92, 95% CI [.89, .94], Factor 1, ϖ = .91, 95% CI [.88, .93], Factor 2, ϖ = .85, 95% CI [.81, .98], and Factor 3, ϖ = .86, 95% CI [.81, .90].

**Table 3:** Exploratory factor analysis of the BAS in Subsample 1

BAS item	MSA	Factor		
		1	2	3
5. Reduced leisure time	.886	.947		
7. Less time for friends	.889	.852		
4. Change personal plans	.923	.731		
6. Upset household routine	.957	.703		
8. Neglected family’s needs	.938	.637		
3. Difficulty concentrating	.937	.499		
2. Missed work/school	.926	.409		
11. Embarrassed	.895		.781	
14. Resented demands	.887		.763	
10. Friction with others	.843		.676	
9. Family friction	.941		.532	
16. Upset about relative’s change	.924		.471	
15. Felt trapped	.954		.452	
13. Guilty for causing illness	.810			.926
12. Guilty for not helping enough	.838			.794
17. Worry about making illness worse	.863			.638
Sum of Square Loadings^a^		4.159	2.948	2.468
Proportion of variance^a^		.219	.155	.130
Cumulative proportion of variance^a^		.219	.374	.504
McDonald’s ϖ		.91	.85	.86

*Note*. *N* = 114. Bartlett’s test: χ^2^_(171)_ = 1096.121, *p* < .000; Chi-squared test: χ^2^_(101)_ = 93.357, *p* = .693. Extraction method: Weighted Least Square. Rotation method: Oblimin. Items 1, 18, and 19 did load < .40. Blanks represent loading < .40. MSA = Measure of Sampling Adequacy (.50 is an acceptable limit for retaining a variable for the EFA; e.g. [41]. Overall MSA = .900

^a^Rotated solution

The factors correlated positively at the .001 level: ρ_(F1-F2)_ = .531, ρ_(F1-F3)_ = .460, and ρ_(F2-F3)_ = .458. The effect sizes of these correlations were between intermediate and strong [6]. Factor 1 was called “Disrupted Activities”, Factor 2 was called “Personal and Social Dysfunction”, and Factor 3 was called “Worry, Guilt, and Being Overwhelmed”. Factor 1 refers to objective burden, whereas Factor 2 and Factor 3 refer to subjective burden.

### Confirmatory factor analysis of the model obtained for the BAS

**Fig. 2:**
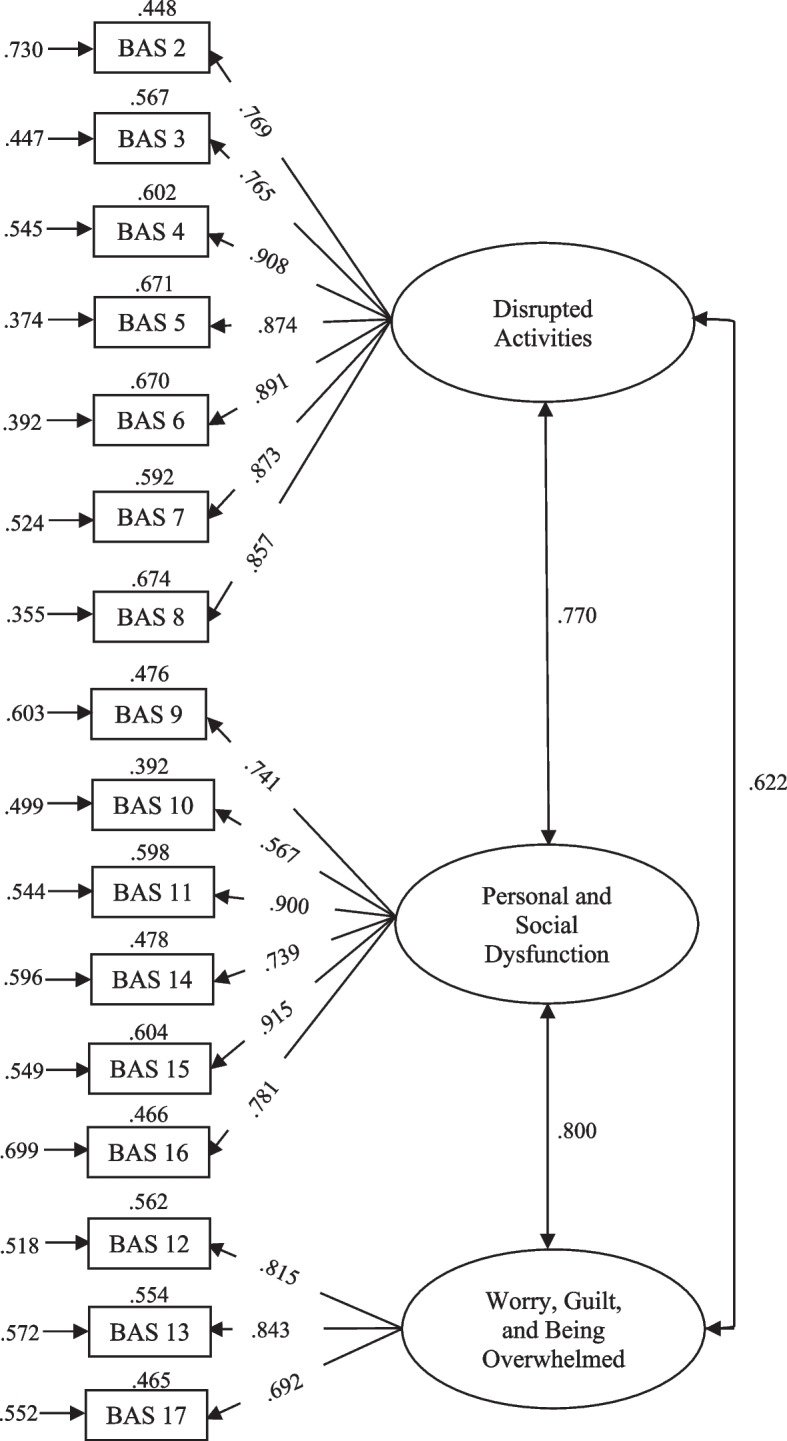
Model for the BAS obtained in the present study. *Note.* Values at the top of each rectangle are *R*^2^; values at the left of each rectangle are errors

The model obtained for the BAS showed an excellent fit: χ^2^_(101)_ = 56.873, *p* = 1.000, CFI = 1.000, TLI = 1.000, RMSEA = .000, 95% CI [.000, .000], SRMR = .060. All the parameters were significant at the .01 level (Fig. 2).

### Concurrent and divergent validity of the model obtained for the BAS

Correlations between the BAS, the MQLI, and the DASS-21 were in the expected direction and had effect sizes that varied between intermediate and strong [6] (Table 4).

**Table 4:** Correlations (Spearman’s ρ) between the BAS, the MQLI, and the DASS-21 scales

Variable	Burden total	Disrupted Activities	Worry, Guilt, and Being Overwhelmed	Personal and Social Dysfunction
Quality of Life	-.405*** I	-.365*** I	-.321*** I	-.384*** I
Anxiety	.500*** S	.419*** I	.486*** I	.441*** I
Depression	.479*** I	.393*** I	.487*** I	.403*** I
Stress	.529*** S	.463*** I	.505*** S	.448*** I

*Note. N* = 233. S = Strong effect size; I = Intermediate effect size [6]

^***^ *p* < .001

## Discussion

This study aimed to test the underlying factors, provide evidence about its internal consistency, and analyze the fit and construct validity of the BAS in a sample of Spanish caregivers of people diagnosed with BPD.

### Structural validity of the model obtained for the BAS

Pioneering work by Horwitz and Reinhard [21] and Reinhard et al. [36] obtained five-factor models for the BAS. As indicated above, these studies failed to meet some of the basic requirements for exploratory factor studies, such as not accepting factors with less than three items or not including the same item in two or more factors. In our opinion, the lack of methodological rigor in these studies suggested the need for a more rigorous analysis of the structure of this scale. Subsequent studies obtained two-factor [17], three-factor [23, 29], four-factor [22, 33], and five-factor [3] models for the BAS.

We obtained a three-factor model for the BAS (Disrupted Activities; Worry, Guilt, and Being Overwhelmed; and Personal and Social Dysfunction) using exploratory procedures, as in Ivarsson et al. [23] (Activity Limitation,Worry and Guilt; and Social Strain) and Kwak et al. [29] (Activity Limitation,Social Strain; and Feelings of Worry and Guilt), with a similar distribution of items per factor, but small differences: in contrast to the studies by Ivarsson et al. [23] and Kwak et al. [29], in our study, item 1 was not included in the disrupted or limited activities factor, item 19 was not included in the social factor, and item 18 was not included in the worry-and-guilt factor.

It is worthy to note that (1) the mean for item 10 (Friction with others) was the lowest, and this item was included in Factor 2 (Personal and Social Dysfunction); (2) the mean score on item 1 (Financial problems) was low; (3) the highest mean score was on item 18 (Worry about the future), close to the maximum of the scale; and (4) the mean for item 19 (Stigma upsetting) was comparable to that of other BAS items that were included in any factor (e.g. items 16 (Upset about relative’s change) and 17 (Worry about making illness worse), among others). With regard to item 1 (Financial problems), its non-inclusion could be due to the fact that financial problems, even if they exist, are not significantly related to caring for a relative with an SMD (e.g. expenses for psychiatric medication, psychotherapy costs, help from external caregivers, among others). Regarding items 18 (Worry about future) and 19 (Stigma upsetting), it is possible that in the past 6 months the family caregivers have not felt a significant amount of worry about the future or upset due to the stigma of having a relative diagnosed with an SMD (it should be noted that the items on the BAS are responded to in relation to the following statement: “Please, would you tell me to what extent you have had any of the following experiences in the past 6 months?”). It would be interesting to investigate how family caregivers interpret worrying about the future: is it concern about possible economic hardship, the evolution of their relative diagnosed with BPD, or the future understood in a vague and diffuse way? Likewise, it would be interesting to find out whether family caregivers are aware of the meaning of the term “stigma” and, therefore, respond appropriately to that item. It must be noted that, in the present study, the sample was exclusively composed of family caregivers of people diagnosed with BPD, unlike previous studies that used samples composed of people with different diagnoses of SMD. The different composition of the samples used in these studies may have led to the differences in the results of the BAS structural analyses.

Previous studies that analyzed the structure of the BAS used Principal Component Analysis (PCA) with Varimax rotation, with the exception of Hunger et al. [22], who used CFA. Both PCA and Varimax assume uncorrelated factors (e.g. [25]). We used an EFA, specifically the Weighted Least Squares extraction method with Oblimin rotation method [15], because we assumed that the factors underlying the BAS items were correlated (like [22]. The assumption that factors are not correlated seems unlikely in the case of psychological variables, such as those measured by the BAS. Objective and subjective burden are correlated aspects or facets of the same burden experience. Therefore, it seems more appropriate to assume that the BAS factors are correlated and use an oblique rotation method (such as Oblimin) rather than an orthogonal method (such as Varimax) in the EFA (e.g. [15]).

We tested the model obtained for the BAS using confirmatory procedures in Subsample 2. Results showed the goodness of this model. Only Hunger et al. [22] tested the BAS structure using confirmatory procedures, although in reality these authors analyzed the models proposed by Reinhard et al. [36], but introducing a new parameter, i.e. correlations between the factors.

In conclusion, the present study offers a cross-analysis, both exploratory and confirmatory, of the BAS, and it proposes a reduced three-factor model with 16 items that shows good structural properties.

### Internal consistency of the model obtained for the BAS

The model for the BAS obtained in this study showed good internal consistency, with estimations between .85 and 0.91 for the factors and .92 for the whole scale, which are similar to those obtained in previous studies that found Cronbach’s alphas between .89 [3, 36] and .92 [22] for the whole BAS. One exception is the study by Hunger et al. [22], who found a Cronbach’s alpha of .64 for the Guilt subscale (which contained the items, 12, 13, and 17,these items were included in the Worry, Guilt, and Being Overwhelmed factor obtained in our study), an alpha of .74 for the Time Perspective subscale, and an alpha of .78 for the Personal Distress subscale.

### Construct validity of the model obtained for the BAS

As expected, the factors in the model for the BAS obtained in this study correlated negatively with quality of life and positively with anxiety, depression, and stress. These results are comparable to those obtained in the study by Kwak et al. [29], and support the construct validity of that scale.

### Limitations of this study and suggestions for future studies

Some limitations of the present study should be mentioned. Regarding the sample, it would be desirable to confirm the structure obtained for the BAS in a larger sample than the one we used. It would also be interesting to analyze the invariance of this scale with regard to sex and other variables of clinical interest. For example, in our study, most of family caregivers of people diagnosed with BPD were biological mothers, and as previous studies have found, this population could be more vulnerable to feelings of burden than other caregivers and, therefore, need more support [26]. It would be interesting to test the BAS invariance between groups of caregivers. Likewise, it would be interesting to analyze whether the caregivers of people diagnosed with BPD suffer some psychological disorder, such as anxiety and depression among others, as a result of their family member’s disorder, in order to analyze its impact in the relationship with her/him and whether it can be a factor of aggravation of the disorder suffered by the relative under their care.

It would be interesting to have repeated measurements in a longitudinal design, in order to analyze the test–retest reliability of the model obtained for the BAS, and to confirm the construct validity of this scale using scales other than the ones used in our study.

It would be useful to take into account the psychological health of family caregivers of people diagnosed with SMD (e.g. [37]), and to know whether they are receiving pharmacological treatment and/or psychotherapy.

Future studies should consider the socio-economic status of the family caregivers, in order to identify factors that might facilitate or hinder the care of a relative diagnosed with an SMD, and assess the possibility of drafting statements that are more in line with the content of each item on the BAS.

It would be interesting to analyze the psychometric properties of the BAS model obtained in our study in family caregivers of people diagnosed with an SMD other than BPD, chronic disabilities or degenerative diseases, among others.

## Conclusion

The BAS can be a useful instrument for clinicians, who can assess burden in family caregivers of people diagnosed with BPD (or another SMD) and improve the efficiency of programs designed to provide resources and develop skills to manage the symptoms of the burden of caring, such as Family Connections (e.g. [10, 11, 20]), and positively influence their mental health and personal well-being (e.g. [1, 9]). Scores on the BAS before and after the program can be a valid and reliable indicator of change in the family caregivers of people with a diagnosis of SMD. The model for the BAS obtained in this study is a valid, reliable, and useful tool for assessing burden in family caregivers of relatives diagnosed with BPD.

## Data Availability

The datasets generated during and/or analysed during the current study are available from 10.7910/DVN/8MU95A (Harvard Dataverse).
